# Electrically tunable dipolar interactions between layer-hybridized excitons†

**DOI:** 10.1039/d3nr01049j

**Published:** 2023-06-13

**Authors:** Daniel Erkensten, Samuel Brem, Raül Perea-Causín, Joakim Hagel, Fedele Tagarelli, Edoardo Lopriore, Andras Kis, Ermin Malic

**Affiliations:** a Department of Physics, Chalmers University of Technology 41296 Gothenburg Sweden daniel.erkensten@chalmers.se ermin.malic@physik.uni-marburg.de; b Department of Physics, Philipps-Universität Marburg 35037 Marburg Germany; c Institute of Electrical and Microengineering, École Polytechnique Fédérale de Lausanne (EPFL) Lausanne Switzerland; d Institute of Materials Science and Engineering, École Polytechnique Fédérale de Lausanne (EPFL) Lausanne Switzerland

## Abstract

Transition-metal dichalcogenide bilayers exhibit a rich exciton landscape including layer-hybridized excitons, *i.e.* excitons which are of partly intra- and interlayer nature. In this work, we study hybrid exciton–exciton interactions in naturally stacked WSe_2_ homobilayers. In these materials, the exciton landscape is electrically tunable such that the low-energy states can be rendered more or less interlayer-like depending on the strength of the external electric field. Based on a microscopic and material-specific many-particle theory, we reveal two intriguing interaction regimes: a low-dipole regime at small electric fields and a high-dipole regime at larger fields, involving interactions between hybrid excitons with a substantially different intra- and interlayer composition in the two regimes. While the low-dipole regime is characterized by weak inter-excitonic interactions between intralayer-like excitons, the high-dipole regime involves mostly interlayer-like excitons which display a strong dipole–dipole repulsion and give rise to large spectral blue-shifts and a highly anomalous diffusion. Overall, our microscopic study sheds light on the remarkable electrical tunability of hybrid exciton–exciton interactions in atomically thin semiconductors and can guide future experimental studies in this growing field of research.

## Abstract 2

Electrical field tuning of hybrid exciton–exciton interactions in WSe_2_ homobilayers.

## Graphical abstract

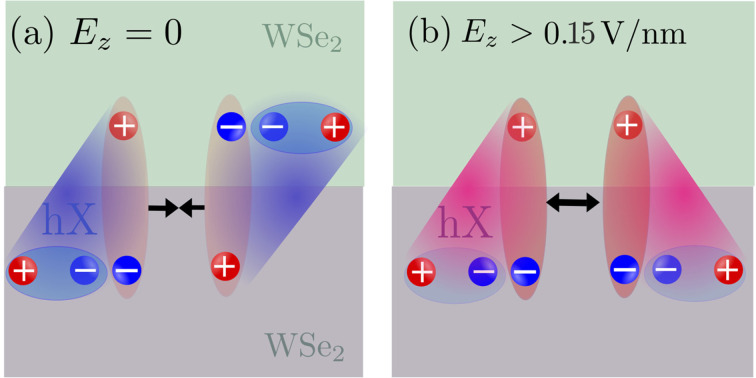

Recently, two-dimensional van der Waals heterostructures, formed by stacking transition-metal dichalcogenide monolayers on top of each other, have emerged as a promising platform for engineering strong correlations, topology and intriguing many-body interactions.^1–5^ In particular, these structures exhibit spatially separated interlayer excitons, *i.e.*, Coulomb-bound electron–hole pairs where the constituent electrons and holes reside in different layers, which display permanent out-of-plane dipole moments.^6–10^ Furthermore, intra- and interlayer exciton states can be efficiently hybridized *via* electron and hole tunneling, and form new hybrid excitons (hX) that inherit properties of both exciton species.^11–14^ The formation of hybrid excitons is particularly favorable in naturally stacked homobilayers, as opposed to type-II heterostructures where the dominating exciton species have mostly interlayer character.^15,16^

Moreover, the ground state of hybrid excitons can be optically inactive or momentum-dark,^17^ as is the case in WSe_2_ homobilayers (Fig. 1(a)).^18,19^ Here, the efficient electron tunneling at the Λ-point of the Brillouin zone (and less efficient hole tunneling at the K-point) results in a strongly hybridized KΛ exciton state.^13^ Furthermore, in naturally stacked H-type (2H) WSe_2_ homobilayers, the KΛ state is energetically degenerate with the K′Λ′ state, however these two states exhibit opposite dipole orientations (as a consequence of the inverted spin–orbit splitting in one of the layers^13^). This stacking configuration also enables the formation of other exciton species, such as KΛ′ excitons, which lie energetically close to the degenerate KΛ and K′Λ′ states. Intriguingly, the KΛ′ exciton state exhibits a much larger interlayer component than the KΛ state as schematically illustrated in Fig. 1(a) (where green and gray bands refer to the upper and lower TMD layer, respectively). As hybrid excitons are partly of interlayer character, they also exhibit an out-of-plane dipole moment which couples to externally applied electric fields *via* the quantum-confined Stark effect,^20–24^ such that the interlayer component of these excitons and even the ordering of different hybrid exciton states can be tuned.^25,26^ This implies that also the interactions, in particular the dipole–dipole repulsion, between different types of hybrid excitons should be electrically tunable. Hence, a remarkable number of fundamentally and technologically relevant phenomena governed by exciton–exciton interactions in TMDs could potentially be electrically controlled. Some of these phenomena include experimentally observed blue-shifts of exciton resonances with excitation power,^6,27^ anomalous exciton transport,^28,29^ and even the stability of Bose–Einstein condensates,^30,31^ the conditions for superfluidity^32–34^ and the exciton compressibility that is important for the characterization of excitonic insulators.^35^

**Fig. 1:**
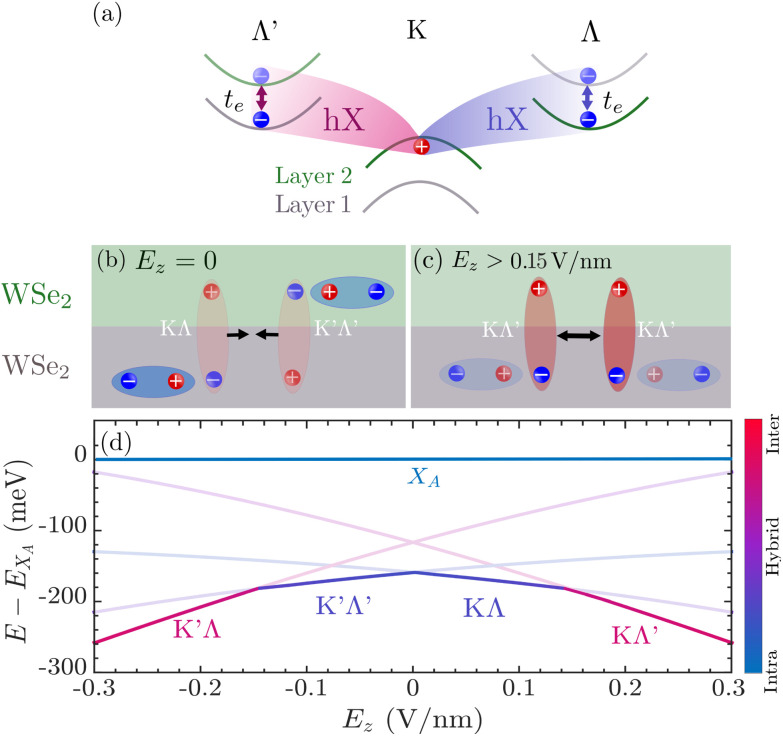
Hybrid exciton species in naturally stacked bilayer WSe_2_. (a) KΛ and KΛ′ hybrid excitons (hX) are primarily formed from electrons and holes in the same or different layers, respectively. The two layers are indicated with green and gray lines. The hybridization is predominantly induced *via* electron (*t*_e_) tunneling. Note that the K′Λ′ and K′Λ excitons, which are degenerate with the respective KΛ and KΛ′ states and exhibit opposite dipole moments, are not shown. (b) At vanishing electric fields the degenerate KΛ and K′Λ′ hX are the energetically lowest states. These are mostly intralayer-like in nature and attract each other due to their opposite dipole moments. (c) At electric fields *E*_*z*_ > 0.15 V nm^−1^, KΛ′ excitons constitute the energetically lowest states. These are mostly interlayer in nature and repel each other. (d) Exciton landscape as a function of electric field, with the colorbar revealing a transition from a mostly intralayer-like (blue) to a mostly interlayer-like exciton state (red) at elevated electric fields. The thick line denotes the energetically lowest state that changes depending on the electric field. The energies are given with respect to the KK intralayer exciton (usually denoted as *X*_A_ in literature).

In this work, we develop a material-specific and predictive many-particle theory of hybrid exciton–exciton interactions using the density matrix formalism. We investigate the impact of electric fields on density-dependent energy renormalizations and exciton transport at elevated excitation densities in naturally stacked WSe_2_ homobilayers. We show that two intriguing interaction regimes emerge when applying an out-of-plane electric field: (i) a low-dipole regime at *E*_*z*_ ≲ 0.15 V nm^−1^ (Fig. 1(b)), where interactions are governed by mostly intralayer-like KΛ and K′Λ′ excitons which mutually attract each other, and (ii) a high-dipole regime at *E*_*z*_ ≳ 0.15 V nm^−1^ (Fig. 1(c)), where mostly interlayer-like KΛ′ excitons constitute the energetically lowest state which exhibits a strong dipole–dipole repulsion. These regimes give rise to substantially different behaviors for the experimentally accessible energy renormalizations and exciton transport. While the low-dipole regime is characterized by negligible exciton line-shifts and a conventional diffusion, the high-dipole regime exhibits considerable blue-shifts of tens of meVs and a highly anomalous diffusion. Overall, our work provides a recipe for future experiments on how to tune the hybrid exciton–exciton interaction and in particular exciton transport at elevated excitation powers.

## Hybrid exciton landscape

To model exciton–exciton interactions between layer-hybridized excitons in TMD bilayers, we first set up an excitonic Hamilton operator *H* = *H*_*x*,0_ + *H*_*x*−*x*_ expressed in a monolayer eigenbasis.^36^ Here, the first part of the Hamiltonian takes into account the centre-of-mass motion of intra- and interlayer excitons, their Coulomb binding, and their hybridization *via* an effective tunneling model. This approach to layer-hybridized excitons has been shown to accurately reproduce the exciton energies and capture well the mixing of intra- and interlayer exciton states in TMD homobilayers as obtained from *ab initio* calculations.^24^ The Hamiltonian which captures exciton hybridization reads^13,37^1
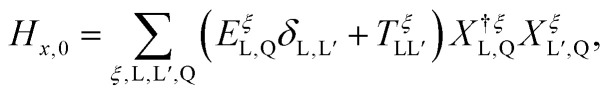
with the first term being the exciton dispersion 
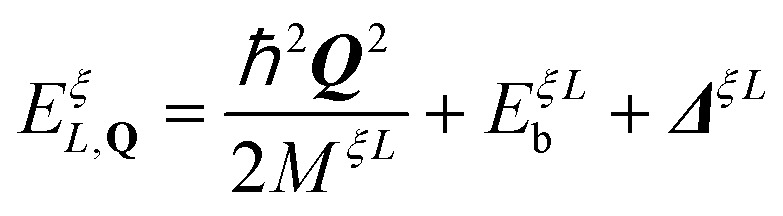
, and the exciton binding energy, *E*^*ξL*^_b_, obtained from solving the bilayer Wannier equation.^19,38^ Here, *M*^*ξL*^ is the total exciton mass, *L* = (*l*_h_,*l*_e_) is a compound layer index, *ξ* = (*ξ*_h_,*ξ*_e_) is the exciton valley and ***Q*** is the centre-of-mass momentum. Furthermore, *Δ*^*ξL*^ contains the valley-specific bandgap. Note that, due to the degeneracy between exciton states with different spin-valley configurations (neglecting electron–hole exchange^39^), it is sufficient to consider a single spin system, *e.g.* exciton states being formed by spin-up valence and conduction bands, so that spin indices can be omitted. The excitonic operators X^†*ξ*^_*L,**Q***_ (X^*ξ*^_*L*,***Q***_) create (annihilate) intralayer (X, *l*_e_ = *l*_h_) or interlayer (IX, *l*_e_ ≠ *l*_h_) excitons. The second part of eqn (1) takes into account the tunneling of electrons and holes between different layers *via* the excitonic tunneling matrix element, *T*^*ξ*^_*LL*′_.^19^ The latter is dependent on electron/hole tunneling strengths and excitonic wave function overlaps, *cf*. ESI section I† for details. By performing the basis transformation 
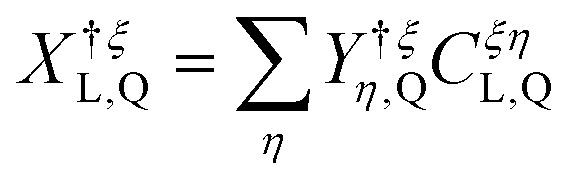
, introducing the *hybrid* exciton operators *Y*^(†)^_*η*_, the hybrid exciton state *η*, and the mixing coefficients *C* determining the relative intra- and interlayer content of the hybrid exciton, the Hamiltonian in eqn (1) is diagonalized and becomes2
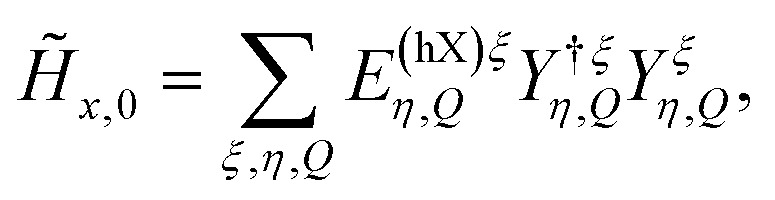
where the hybrid exciton dispersion *E*^(hX)*ξ*^_*η*,***Q***_ along with the mixing coefficients are obtained from solving the hybrid exciton eigenvalue problem, *cf*. ESI section I.† In this work, we are only concerned with the lowest hybrid exciton state *η*, and omit this index in the following. In Fig. 1(d), we show the hybrid exciton landscape for an hBN-encapsulated and naturally stacked (2H) WSe_2_ homobilayer, including the four lowest-lying states. In Table S1 in ESI section I,† we include also higher-lying transitions. The hybrid exciton eigenenergy *E* ≡ *E*^(hX)^_***Q***=0_ is given relative to the intralayer A exciton energy, *E*_*X*_A__. We find that *ξ* = (*ξ*_h_,*ξ*_e_) = KΛ/KΛ′ exciton states constitute the energetically lowest states, lying approximately 160 meV below the bright *X*_A_ state. The colorbar indicates the corresponding interlayer component of the mixing coefficient, revealing the hybrid nature of KΛ/K′Λ′ excitons, *cf*. Fig. 1(d) at *E*_*z*_ = 0. Note that the exciton ground state in the considered material as predicted by our microscopic model has also been obtained in recent GW-BSE many-body calculations.^18^ Moreover, it was also recently experimentally verified, *via* magnetoluminescence measurements, that the *g*-factors extracted from low-energy peaks in the PL spectrum in WSe_2_ are consistent with theoretically predicted *g*-factors for KΛ transitions.^40,41^

Furthermore, we study the exciton landscape as a function of an out-of-plane electric field, *E*_*z*_. This is done by exploiting the electrostatic Stark shifts of the interlayer exciton energies, which influence the intra- and interlayer composition of hybrid excitons.^20,22,24^ Intriguingly, we find that for positive (negative) electric fields |*E*_*z*_| > 0.15 V nm^−1^, the energetically lowest state corresponds to the KΛ′ (K′Λ) state, *i.e.* the ordering of different exciton states is changed. This is explained by the fact that, as a consequence of the band-ordering (Fig. 1(a)), the KΛ′ state carries a significantly larger interlayer component than the KΛ state and is as such easier modulated with respect to electric fields. In particular, the KΛ′ and KΛ states possess an interlayer component |*C*_IX_|^2^ of 0.64 (0.80) and 0.23 (0.39), respectively, at *E*_*z*_ = 0(0.3) V nm^−1^. The fact that the dominating exciton species at elevated electric fields carries a large interlayer component and consequently a large dipole moment is also reflected in the stronger exciton–exciton interaction, as we shall see in the following.

## Hybrid exciton–exciton interactions

Now, we consider the interacting part of the Hamiltonian, *H*_*x*−*x*_. In this work, we focus on the direct part of the interaction, and the contributions from interlayer excitons. Interlayer exchange interactions are seen to give a minor correction to the direct dipole–dipole interaction.^42,43^ Although intralayer exchange interactions (taking into account exchange of individual carriers) are dominant in TMD monolayers,^44,44,45^ they are known to have a negligible impact on experimentally accessible density-dependent energy renormalizations, as their contributions are largely cancelled out against contributions due to higher-order correlation effects.^46,47^ This is also supported by recent experiments, which report negligible shifts with excitation power of intralayer exciton resonances and sizable blue-shifts in luminescence spectra for interlayer excitons.^6,28^ Furthermore, we assumed that the excitons can be treated as independent bosons, which holds in the weakly interacting limit *n*_*x*_*a*^2^_B_ ≪ 1, where *a*_B_ is the exciton Bohr radius and *n*_*x*_ is the exciton density.^48^

We transform the interaction Hamiltonian to the hybrid basis (*cf.* ESI section II† for details) resulting in3
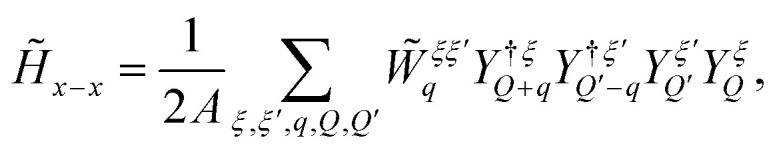
with the hybrid dipole–dipole interaction matrix element 
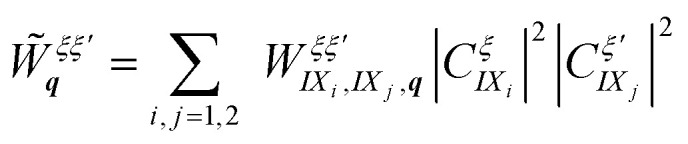
 and the normalization area *A*. The hybrid exciton–exciton interaction crucially includes the pure interlayer dipole–dipole interaction between different interlayer exciton species IX_*i*_, *i* = 1, 2, weighted by the corresponding mixing coefficients. The interlayer dipole–dipole matrix element reads in the long wavelength limit 
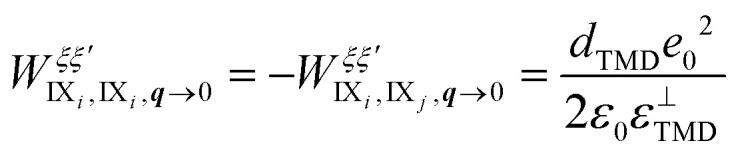
, where *i* ≠ *j* and with *d*_TMD_ and *ε*^⊥^_TMD_ being the TMD thickness and the out-of-plane component of the dielectric tensor of the TMD, respectively. The sign difference between the interactions is a consequence of the opposite dipole orientations of the interlayer excitons IX_1_ and IX_2_ (*cf.*Fig. 1(b)). The full hybrid Hamiltonian including intra- and interlayer direct and exchange contributions is derived in ESI section II.†

**Fig. 2:**
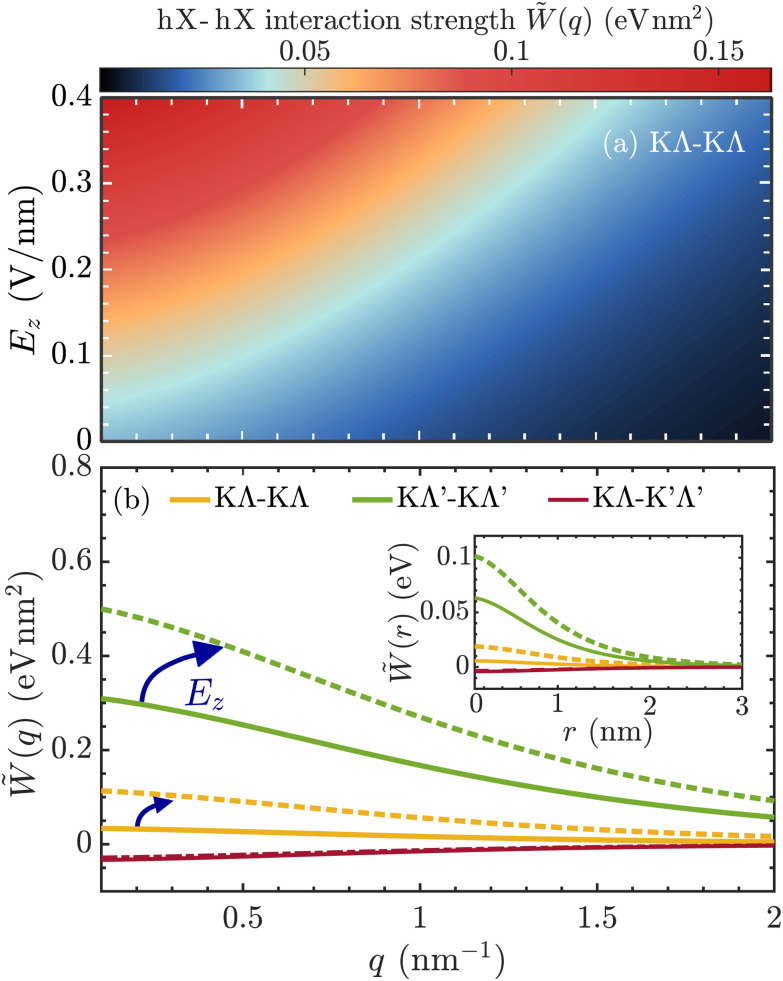
Hybrid exciton–exciton interactions. (a) Momentum- and electric-field dependent interaction strength involving KΛ hX. The interaction strength increases as the interlayer component is enhanced under the application of an out-of-plane electric field, *E*_*z*_. (b) Momentum-dependent interaction strengths for different electric fields and between different types of hybrid excitons. Solid lines correspond to the case *E*_*z*_ = 0 and dashed lines to the case of *E*_*z*_ = 0.3 V nm^−1^. The inset shows the corresponding real-space interaction, *W̃*(*r*).

In Fig. 2(a), we display the hybrid exciton–exciton interaction matrix element for *ξ* = *ξ*′ = KΛ hybrid excitons as a function of momentum and out-of-plane electric field. The interaction is repulsive (>0) and maximized in the long wavelength limit. The interaction strength is also found to be highly tunable with respect to electric fields *via* its quartic dependence on interlayer mixing coefficients (*cf.*eqn (3)). In particular, the interlayer component of hybrid excitons is enhanced with *E*_*z*_, if the electric field is applied parallel to the dipole moment of the hX, *cf*. Fig. 1(c). In Fig. 2(b), we consider interactions between different types of hybrid exciton species at vanishing electric fields (solid lines) and at *E*_*z*_ = 0.3 V nm^−1^ (dashed lines). Due to their large interlayer component (Fig. 1(d)), KΛ′ excitons exhibit the strongest dipole–dipole repulsion, which is further enhanced with *E*_*z*_. Furthermore, we note that the interaction between KΛ and K′Λ′ excitons is attractive (<0), *cf*. Fig. 1(b). This is a consequence of the interlayer component of these excitons having opposite dipole moments. As such, the exciton states energetically shift in opposite directions under the application of an electric field and the increase of interlayer component in one of the excitons is compensated by a decrease of interlayer component in the other exciton (Fig. 1(d)). This yields an interaction strength which is largely independent on electric field. We also show the hybrid exciton–exciton interaction in real space (inset in Fig. 2(b)), and identify the dipole–dipole-like character of the interaction between excitons of the same valley species at large distances, *i.e. W̃*(*r*) ∼ *d* ^2^_TMD_/*r*^3^ (*cf.* ESI section III†).^49,50^ Finally, we note that the real-space exciton–exciton interaction, crucially including the dipole–dipole interaction, is a key ingredient in the Bose–Hubbard model, which can be exploited to investigate the conditions for different quantum phases of excitonic systems, such as superfluidity, in semiconductor moiré materials.^32,33,51^

Having microscopic access to the hybrid exciton–exciton interaction matrix elements enables us to study density-dependent energy renormalizations observable in photoluminescence (PL) spectra. In particular, given the large electrical tunability of exciton–exciton interactions, we expect that applying electric fields in combination with increasing pump power can be used to engineer substantial blue-shifts of exciton luminescence peaks. This offers an intriguing way of realizing strong many-body interactions in atomically thin semiconductors. Note that the relevant excitons in homobilayer WSe_2_ are momentum-dark (Fig. 1(a)), and become only visible *via* phonon sidebands in low-temperature PL.^22,26,52–54^ In our theoretical model, we derive the density-dependent energy renormalization δ*E*^*ξ*^ by evaluating the Heisenberg equation of motion for the hybrid polarization on a Hartree–Fock (mean-field) level and find4
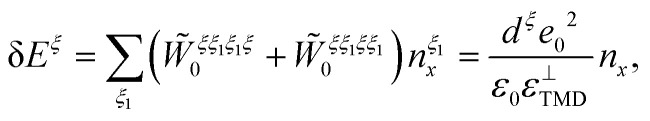
where the first term reflects direct exciton–exciton interactions and the second term is due to exciton exchange,^55^ with the interaction matrix element 
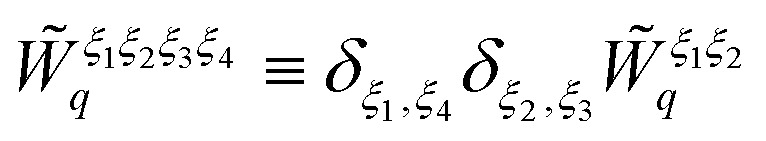
 (*cf.*eqn (3)). The interaction matrix elements are evaluated in the long wavelength limit, such that the energy renormalization becomes momentum-independent. This is well justified when the exciton distribution is strongly peaked around small centre-of-mass momenta, *i.e.* at lower temperatures. The interaction strength is weighted by the valley-specific hybrid exciton density 
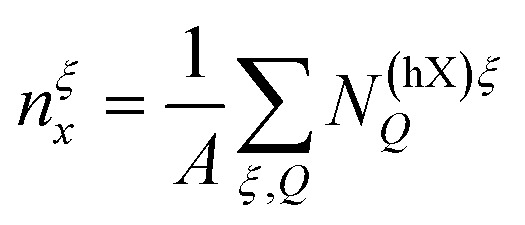
, and the exciton occupation is estimated by a thermalized Boltzmann distribution *N*^(hX)*ξ*^_***Q***_ ∼ *n*_*x*_ exp(−*E*^(hX)*ξ*^_***Q***_/(*k*_B_*T*)) such that the energy renormalization scales linearly with the total exciton density 
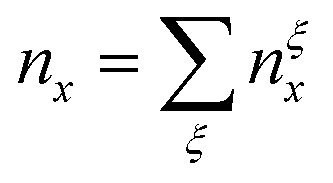
. This allows us to absorb the exciton–exciton interaction strength and relative occupations in an effective valley-dependent dipole length *d*^*ξ*^. In this way, the energy renormalization of a single exciton species *ξ* is completely characterized by its effective dipole length. A detailed derivation of eqn (4) and the relevant (electric field-dependent) valley-specific dipole lengths are found in ESI section IV.†

**Fig. 3:**
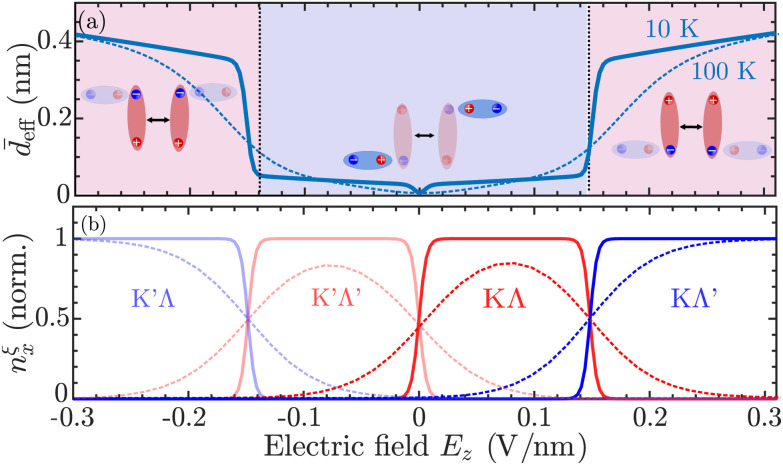
Dipole length of hybrid excitons. (a) Average dipole length *d̄*_eff_ as a function of electric field revealing a drastic increase of the dipole length for |*E*_*z*_| > 0.15 V nm^−1^ at low temperatures (*T* = 10 K). This is explained by a large valley-specific dipole length *d*^*ξ*^_eff_ of mostly interlayer-like *ξ* = KΛ′/K′Λ excitons and a predominant occupation *n^ξ^_x_* of these excitons at elevated electric fields shown in (b)). The average dipole length and valley occupations at *T* = 100 K are shown as dashed lines.

Furthermore, we define an *average* effective dipole length of the exciton gas, 
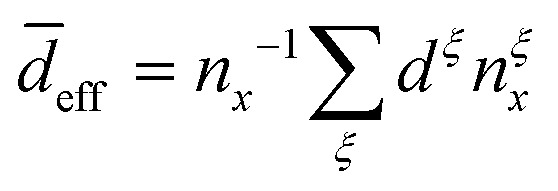
. This quantity is crucial to access macroscopic transport properties, as further discussed in the next section. The average effective dipole length is presented in Fig. 3(a) as function of electric field, together with the normalized valley-specific exciton density in Fig. 3(b). At low electric fields, the exciton occupation is shared between the energetically degenerate KΛ and K′Λ′ states (*cf.* also Fig. 1(d)). These excitons interact weakly *via* dipolar interactions and combined with their attractive mutual interaction this results in suppressed average effective dipole lengths, *d̄*_eff_ ≈ 0.01 nm. In contrast, at elevated electric fields |*E*_*z*_| > 0.15 V nm^−1^, KΛ′/K′Λ excitons are found to give rise to large effective dipole lengths *d̄*_eff_ ≈ 0.4 nm, reflecting their large occupation (Fig. 3(b)) as well as their large interaction strength (Fig. 2(b)). Here, we remark that the extracted effective dipole length at large electric fields can be compared with the dipole length of a pure interlayer exciton, *d*_IX_, here assumed to be equal to the TMD layer thickness, *d*_TMD_ = 0.65 nm (ESI section I†). In particular, it holds that *d̄*_eff_ = *d*_IX_|*C*^KΛ′^_IX_|^4^ ≈ 0.4 nm, *i.e.* the effective dipole length is obtained by weighting the pure interlayer exciton dipole length by the interlayer component of the mixing coefficient. Furthermore, we note that the transition between the low-dipole regime in which KΛ and K′Λ′ excitons are prevalent and the high-dipole regime dominated by KΛ′ or K′Λ excitons can be tuned by raising the temperature, *cf*. the dashed curve in Fig. 3(a) displaying the average effective dipole length at *T* = 100 K. This is a consequence of intralayer-like and interlayer-like exciton states being simultaneously populated at high temperatures. Considering the case of *T* = 100 K, there exists a sizable occupation of KΛ/K′Λ′ excitons at finite electric fields and a large electric field (|*E*_*z*_| ≈ 0.3 V nm^−1^) is therefore required for the high-dipole (KΛ′/K′Λ) regime to be reached (*cf.* dashed lines in Fig. 3(b)).

## Anomalous hybrid exciton transport

For a spatially dependent exciton density *n*_*x*_ → *n*(***r***), the density-dependent energy renormalization due to repulsive exciton–exciton interactions gives rise to a drift force −∇(δ*E*(***r***)). The latter drags excitons away from the excitation spot^28,29,56^ in analogy to exciton funneling in strain potentials.^57^ This can be described by the two-dimensional drift-diffusion equation for the exciton density:5
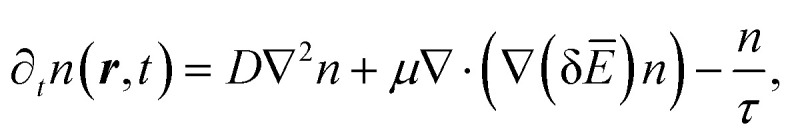
which is derived using the Wigner function formalism.^43,58^ Here, D is the diffusion coefficient governing the free propagation of excitons, 
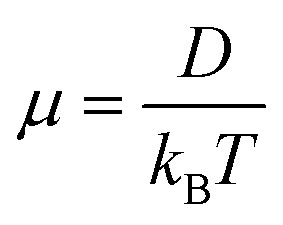
 is the exciton mobility, *T* being temperature and *τ* is the exciton life-time. The second term in eqn (5) is the drift-term dictated by the averaged energy renormalization 
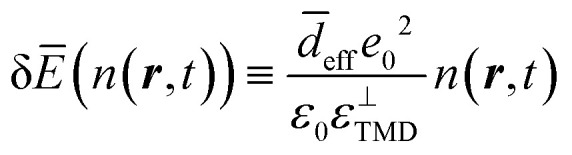
, in which the average effective dipole length *d̄*_eff_ crucially enters (Fig. 3(a)). Here, we made use of the fact that the length-scale of the exciton–exciton interaction is much smaller than the spatial variations of the exciton density. While the former is of the order of 1 nm (*cf.* inset in Fig. 2(b)), the latter is typically in the μm range in experiments.^29^ In order to arrive at eqn (5), we assumed that all exciton states *ξ* which contribute to the total exciton population 
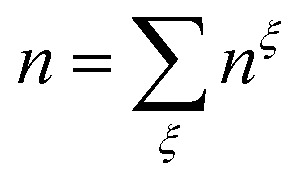
 diffuse with the same diffusion coefficient *D*^*ξ*^ ≈ *D* and that the total population is in thermal equilibrium with the lattice. The first assumption is reasonable since the diffusion coefficient is mainly determined by the effective exciton mass, which is the same in the considered states. The slow thermal equilibration of the exciton gas at low temperatures can, in principle, influence the diffusion dynamics,^59^ but these effects have not been observed for the exciton diffusion in van der Waals heterostructures even at cryogenic temperatures.^29^

We now make use of the strong tunability of the dipole length to show that also the exciton transport can be tuned with respect to electric fields. By numerically solving eqn (5) we obtain a microscopic access to the spatiotemporal dynamics of excitons. We initialize the exciton density as a typical Gaussian-shaped laser pulse, *i.e. n*(*x*,*y*,0) = *n*_0_ exp(−(*x*^2^ + *y*^2^)/*σ*_0_^2^) with the initial spot size *σ*_0_^2^ = 1 μm^2^ and set the temperature *T* = 10 K. The initial exciton density is set to *n*_0_ = 10^12^ cm^−2^, such that the drift due to exciton–exciton interactions becomes important and Boltzmann distributions can be used to model the spatiotemporal dynamics of excitons.^43^ The considered initial exciton density is below the exciton Mott transition, which is estimated to occur at densities ∼7 × 10^12^ cm^−2^ in WSe_2_ homobilayers^60^ and we neglect the impact of free carriers on the transport.^61^ Moreover, we assume the diffusion coefficient *D* = 0.3 cm^2^ s^−1^ and the exciton life-time *τ* = 500 ps as obtained from a recent experiment on the same homobilayer.^62^ Note that there is no moiré potential which could trap excitons and slow down their propagation,^28,63–65^ as we are considering untwisted homobilayers with no lattice mismatch.

**Fig. 4:**
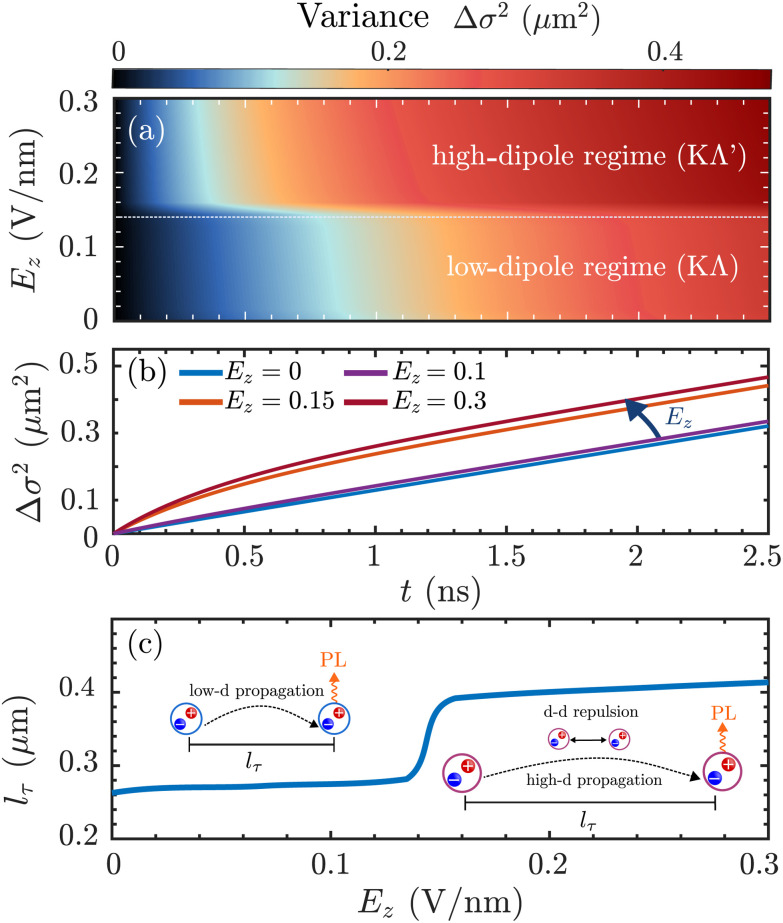
Tunability of hybrid exciton diffusion. (a) Electric-field dependent and time-dependent variances Δ*σ*^2^ = *σ*_*t*_^2^ − *σ*_0_^2^ for the exciton density *n*(*r*,*t*), revealing highly anomalous diffusion for electric fields *E*_*z*_ > 0.15 V nm^−1^ at *T* = 10 K. The dashed white line indicates the transition from the low-dipole regime (dominated by KΛ excitons) to the high-dipole regime (dominated by KΛ′ excitons). (b) Time-dependent variances for different electric fields. For low electric fields (*E*_*z*_ ≤ 0.15 V nm^−1^) conventional diffusion is observed, while at higher electric fields the variance varies non-linearly with time—a hallmark of anomalous diffusion. (c) Diffusion length *l*_*τ*_ as a function of electric field. At large electric fields strong dipole–dipole repulsion between KΛ′ excitons is present, resulting in significantly increased diffusion lengths.

In Fig. 4(a), the time- and electric-field dependent variance Δ*σ*^2^ = *σ*_*t*_^2^ − *σ*_0_^2^ is shown for the case of naturally stacked hBN-encapsulated WSe_2_ homobilayers, revealing a significant broadening of the exciton spatial distribution at electric fields *E*_*z*_ > 0.15 V nm^−1^, corresponding to the high-dipole regime (*cf.*Fig. 3(a)). The transition from the low-dipole to the high-dipole regime results in highly non-linear exciton transport, *cf*. Fig. 4(b). In the low-dipole regime (Fig. 3(a)), excitons are not affected by any drift and the width of the distribution varies approximately linearly with time, *i.e.* Δ*σ*^2^ = 4*Dt* according to Fick's law. In contrast, in the high-dipole regime, the exciton drift is highly efficient leading to a super-linear dependence on the variance with respect to time—a hallmark of anomalous diffusion.^28^ Finally, given the fully time-resolved broadening of the exciton distribution, we extract a time-independent measure of the exciton transport, *i.e.* the experimentally tractable diffusion length 
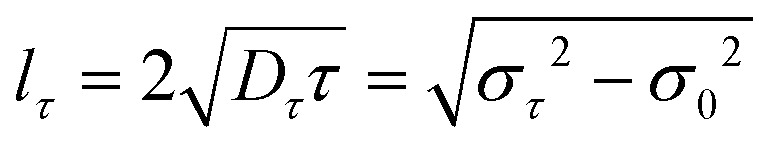
.^66^ This quantity is a measure for how far away from the excitation spot the excitons propagate before recombining and should thus be enhanced with the exciton drift due to dipole–dipole repulsion (Fig. 4(c)). We obtain diffusion lengths in the submicrometer range, concretely 0.25 μm and 0.40 μm in the low- and high-dipole regime, respectively, which are similar to diffusion lengths obtained from previous transport measurements on TMD monolayers and bilayers.^63,67^ We note that excitons in MoSe_2_/hBN/WSe_2_ heterostructures have been reported to exhibit longer diffusion lengths of 1–2 μm,^29^ since excitons in these structures are of purely interlayer character and exhibit enhanced dipole moments due to the hBN spacer. Overall, we reveal a remarkable tunability of the diffusion length in the considered WSe_2_ bilayers with electric fields and we find that the transport of hybrid excitons can be electrically controlled, which is of importance for the realization of exciton-based optoelectronic devices.^68,69^

## Conclusions

Our work sheds light on the impact of electric fields on hybrid exciton–exciton interactions in transition-metal dichalcogenide bilayers. We highlight the presence of hybrid excitons in naturally-stacked WSe_2_ homobilayers and find that the energetically lowest exciton state in these structures can be tuned by applying an out-of-plane electric field. The nature of the lowest state varies from mostly intralayer to mostly interlayer character, resulting in a low-dipole and a high-dipole regime at small and large electric fields, respectively. The latter is characterized by strong interactions between hybrid excitons due to the efficient dipole–dipole repulsion. The electrical tunability of the interactions has also direct consequences on exciton transport. In particular, we predict that the transition from low- to high-dipole regime is accompanied by the emergence of anomalous exciton diffusion, which is a characteristic fingerprint of strong dipole–dipole repulsion. The insights obtained from our material-specific and predictive many-particle theory can be used to guide experiments to measure the tunability of hybrid exciton–exciton interactions in atomically thin semiconductors. Furthermore, our study provides tools for investigating the impact of electrically tunable exciton–exciton interactions on other exotic phenomena in semiconductor moiré materials such as exciton condensation and superfluidity.

## Conflicts of interest

There are no conflicts to declare.

## Supplementary Material

NR-015-D3NR01049J-s001
