# Supplementary material for: Electrically tunable dipolar interactions between layer-hybridized excitons
Source: Nanoscale. 2023 Jun 13;15(26):11064–71. doi: 10.1039/d3nr01049j (PMC10324325; doi:10.1039/d3nr01049j)
Supplement: NR-015-D3NR01049J-s001 [file NR-015-D3NR01049J-s001.pdf]

Electronic Supplementary Material (ESI) for Nanoscale.
This journal is © The Royal Society of Chemistry 2023

Supplementary Material for Electrically tunable dipolar interactions between layer-hybridized excitons

Daniel Erkensten[1], Samuel Brem[2], Raül Perea-Causín[1], Joakim Hagel[1], Fedele Tagarelli[3], Edoardo Lopriore[3], Andras Kis[3] and Ermin Malic[2,1]

[1] *Department of Physics, Chalmers University of Technology, 41296 Gothenburg, Sweden*
[2] *Department of Physics, Philipps-Universität Marburg, 35037 Marburg, Germany*
[3] *Institute of Electrical and Microengineering, École Polytechnique Fédérale de Lausanne (EPFL), Lausanne, Switzerland*

I. HYBRID EXCITON LANDSCAPE IN TMD BILAYERS

In here, we discuss how the exciton landscape in TMD bilayers is obtained within our theoretical framework, taking into account the effect of layer-hybridization. The starting-point is a two-dimensional system containing pure intra- and interlayer excitons. The intra- and interlayer exciton binding energies and wave functions in TMD bilayers are obtained from solving the bilayer Wannier equation [1]

$$\frac{\hbar^2\boldsymbol{k}^2}{2m_{\text{red}}^{\xi L}}\varphi_{n,\boldsymbol{k}}^{\xi L}-\sum_{\boldsymbol{q}}V_{\boldsymbol{q}}^{c_{l_e}v_{l_h}}\varphi_{n,\boldsymbol{k}+\boldsymbol{q}}^{\xi L}=E_{n,\text{bind}}^{\xi L}\varphi_{n,\boldsymbol{k}}^{\xi L}\ , \tag{S1}$$

where $\varphi_{n,\boldsymbol{k}}^{\xi L}$ is the excitonic wave function in state $n = 1s, 2s...$, valley $\xi = (\xi_e, \xi_h)$, and layer $L = (l_e, l_h)$ and $E_{n,\text{bind}}^{\xi L}$ is the exciton binding energy. Here, the reduced exciton mass $m_{\text{red}}^{\xi L} = \frac{m^{\xi_e l_e} m^{\xi_h l_h}}{m^{\xi_h l_h}+m^{\xi_e l_e}}$, as well as the screened electron-hole Coulomb interaction $V_{\boldsymbol{q}}^{c_{l_e}v_{l_h}}$ enter. The valley-specific electron (hole) masses $m^{\xi_e l_e}$ ($m^{\xi_h l_h}$) are obtained from DFT calculations [2]. When evaluating the Coulomb matrix elements we explicitly include the finite thickness of the TMD layers as well as the as the dielectric environment via a generalized Keldysh screening [3]. In this work, we explicitly include hybridization of intra (X)- and interlayer (IX) excitons. In particular, the four possible intra- and interlayer exciton states (here expressed as $L \equiv IX_1, IX_2, X_1, X_2$ focusing on the energetically lowest $n = 1s$ transitions such that the exciton index can be omitted) are generally coupled by electron/hole tunneling. The resulting hybrid exciton states are obtained from diagonalizing the following exciton Hamiltonian [4, 5]

$$H_{x,0}=\sum_{\xi,L,\boldsymbol{Q}}E_{L,\boldsymbol{Q}}^{\xi}X_{L,\boldsymbol{Q}}^{\dagger\xi}X_{L,\boldsymbol{Q}}^{\xi}+\sum_{\xi,L,L',\boldsymbol{Q}}T_{LL'}^{\xi}X_{L,\boldsymbol{Q}}^{\dagger\xi}X_{L',\boldsymbol{Q}}^{\xi}\ , \tag{S2}$$

containing the exciton centre-of-mass dispersion $E_{L,\boldsymbol{Q}}^{\xi} = \frac{\hbar^2\boldsymbol{Q}^2}{2M^{\xi L}} + E_{\text{bind}}^{\xi L} + \Delta^{\xi L}$, $M^{\xi L} = m^{\xi_h l_h} + m^{\xi_e l_e}$ being the total exciton mass, $X^{(\dagger)}$ being excitonic and bosonic ladder operators and $\Delta^{\xi L}$ is the valley-specific band gap. The free Hamiltonian also contains a tunneling contribution which takes into account the tunneling of electrons and holes between different layers ($l_e \neq l'_e$ or $l_h \neq l'_h$) via the excitonic tunneling matrix element

$$T_{LL'}^{\xi}=F_{LL'}^{\xi}[T_{l_e l'_e}^{c\xi_e}\delta_{l_h,l'_h}(1-\delta_{l'_e,l_e})-T_{l_h l'_h}^{v\xi_h}\delta_{l_e,l'_e}(1-\delta_{l'_h,l_h})]\ . \tag{S3}$$

The excitonic tunneling matrix element crucially depends on electron and hole tunneling strengths, $T_{l_e l'_e}^{c\xi_e}$ and $T_{l_h l'_h}^{v\xi_h}$ respectively, as well as exciton wave function overlaps $F_{LL'}^{\xi} = \sum_{\boldsymbol{k}}\varphi_{\boldsymbol{k}}^{*\xi L}\varphi_{\boldsymbol{k}}^{\xi L'}$. The electron and hole tunneling strengths are obtained from *ab-initio* calculations and are reported in Ref. [4] for common TMD bilayers. For the considered case of 2H-stacked WSe_2 homobilayers we adopt the tunneling strengths $T^{cK} = 0$, $T^{vK} = 66.9$ meV and $T^{c\Lambda} = 236.6$ meV for the most relevant K/K' and Λ/Λ' valleys in this structure. Note that the electronic tunneling matrix elements are generally stacking- and momentum-dependent, however in this work we focus on naturally stacked (H_h^h) homobilayers, and evaluate the matrix elements at the high-symmetry points. The Hamiltonian in Eq. (S2) is now diagonalized via the basis transformation [5]

$$X_{L,\boldsymbol{Q}}^{\dagger\xi}=\sum_{\eta}C_{L}^{\xi\eta}(\boldsymbol{Q})Y_{\eta,\boldsymbol{Q}}^{\dagger\xi}\ , \tag{S4}$$

where $Y^{(\dagger)}$ is a new set of *hybrid* exciton operators and $C_L^{\xi\eta}(\boldsymbol{Q})$ is the *mixing coefficient* determining the relative intra/interlayer content of the hybrid exciton, enabling us to define a hybrid exciton state as $|hX_\eta\rangle = \sum_{i=1,2}(C_{X_i}^{\eta}|X_i\rangle +$

$C^{\eta}_{IX_i}|IX_i\rangle$) with $\sum_{i=1,2}(|C^{\eta}_{X_i}|^2 + |C^{\eta}_{IX_i}|^2) = 1$ for a fixed hybrid exciton state η. The mixing coefficients are obtained from solving the following hybrid eigenvalue problem[6]

$$E^{\xi}_{L,\boldsymbol{Q}} C^{\xi\eta}_{L}(\boldsymbol{Q}) + \sum_{L'} T^{\xi}_{LL'} C^{\xi\eta}_{L'}(\boldsymbol{Q}) = E^{(hX)\xi}_{\eta,\boldsymbol{Q}} C^{\xi\eta}_{L}(\boldsymbol{Q}) \ , \tag{S5}$$

introducing the hybrid exciton eigenenergy $E^{(hX)\xi}_{\eta,\boldsymbol{Q}}$. We can now express the exciton Hamiltonian above in the hybrid basis such that $H_{x,0} \to \tilde{H}_{x,0}$ with

$$\tilde{H}_{x,0} = \sum_{\xi,\eta,Q} E^{(hX)\xi}_{\eta,\boldsymbol{Q}} Y^{\dagger\xi}_{\eta,\boldsymbol{Q}} Y^{\xi}_{\eta,\boldsymbol{Q}} \ . \tag{S6}$$

By solving the eigenvalue problem in Eq. (S5) we get microscopic access to the full hybrid exciton landscape in TMD bilayers. Furthermore, we investigate the impact of an electric field on the hybrid exciton landscape. This is done by exploiting the electrostatic Stark shift of interlayer resonances, i.e. by taking $E^{\xi}_{L=IX,\boldsymbol{Q}} \to E^{\xi}_{L=IX,\boldsymbol{Q}} + \Delta E$, with $\Delta E = \pm d e_0 E_z$, where $d \approx 0.65$ nm is the dipole length (here assumed to be the same as the TMD layer thickness [7]),e_0 is the electric charge and E_z is the out-of-plane electric field [8]. In this way, hybrid exciton eigenenergies and mixing coefficients become tunable with respect to electric fields. In Table S1, we report the exciton hybrid energies and intralayer and interlayer mixing coefficients for hybrid excitons composed by electrons in the ξ_e = K, K', Λ, Λ' valleys and holes in the ξ_h = K, K' valleys in 2H-stacked hBN-encapsulated WSe_2 homobilayers. The energies are given relative to the intralayer A exciton energy and the electric fields $E_z = 0, \pm 0.3$ V/nm are considered.

Exciton	Energy $E - E_{X_A}$ (meV)			Intralayer component $\lvert C_X\rvert^2$			Interlayer component $\lvert C_{IX}\rvert^2$		
$\xi = (\xi^h, \xi^e)$	$E_z = -0.3$	$E_z = 0$	$E_z = 0.3$	$E_z = -0.3$	$E_z = 0$	$E_z = 0.3$	$E_z = -0.3$	$E_z = 0$	$E_z = 0.3$
KΛ	-123	**-159**	-209	0.85	**0.77**	0.61	0.15	**0.23**	0.39
K'Λ'	-209	**-159**	-123	0.61	**0.77**	0.85	0.39	**0.23**	0.15
KΛ'	-12	-117	**-258**	0.58	0.36	**0.2**	0.42	0.64	**0.8**
K'Λ	**-258**	-117	-12	**0.2**	0.36	0.58	**0.8**	0.64	0.42
KK	-5	0	-179	0.96	1	0.01	0.04	0	0.99
K'K'	-179	0	-5	0.01	1	0.96	0.99	0	0.04
KK'	-58	-53	-137	0.96	1	0.01	0.04	0	0.99
K'K	-137	-53	-58	0.01	1	0.96	0.99	0	0.04

TABLE S1. Exciton landscape in hBN-encapsulated 2H-stacked WSe_2 homobilayers. We provide the valley-specific energies, intralayer components and interlayer components of hybrid excitons for three different values on the electric field, $E_z = 0, \pm 0.3$ V/nm. The energetically lowest transitions for each electric field are marked in bold and energies are given relative to the KK intralayer A exciton energy (E_{X_A}). For vanishing electric fields we find that the KΛ and K'Λ' states represent the energetically lowest states.

Note that the KΛ and K'Λ' exciton states are energetically degenerate at vanishing electric fields. This is a consequence of the H-type stacking, where the individual TMD layers are rotated 180 degrees with respect to each other such that the spin-orbit coupling in one of the layers is inverted. Moreover, these states can be expressed as $|K\Lambda\rangle = C^{K\Lambda}_{X_1}|X_1\rangle + C^{K\Lambda}_{IX_1}|IX_1\rangle$ and $|K'\Lambda'\rangle = C^{K'\Lambda'}_{X_2}|X_2\rangle + C^{K'\Lambda'}_{IX_2}|IX_2\rangle$ such that each of the states only mixes contributions from a single intralayer and a single interlayer exciton species. Hence, it follows that the KΛ and K'Λ' hX carry opposite out-of-plane dipole moments via their interlayer components, and therefore the energy of these states shifts in opposite directions under the application of an electric field (cf. Table S1).

II. HYBRID EXCITON-EXCITON INTERACTION HAMILTONIAN

Here, we provide a microscopic derivation of the hybrid exciton-exciton interaction Hamiltonian. The starting-point is the bilayer carrier-carrier Hamiltonian:

$$H_{c-c} = \frac{1}{2} \sum_{\lambda^{(\prime)},\xi^{(\prime)},l^{(\prime)}} V_{\boldsymbol{q}}^{\lambda_l \lambda'_{l'}} \lambda^\dagger_{\xi,l,\boldsymbol{k}+\boldsymbol{q}} \lambda'^\dagger_{\xi',l',\boldsymbol{k}'-\boldsymbol{q}} \lambda'_{\xi',l',\boldsymbol{k}'} \lambda_{\xi,l,\boldsymbol{k}} , \tag{S7}$$

where $\lambda^{(\prime)} = (c, v)$, ξ, and $l^{(\prime)}$ are the band, valley, and layer indices, respectively. Here, the operators $\lambda^{(\dagger)}$ annihilate (create) carriers in band λ. Moreover, we note that $V_{\boldsymbol{q}}^{\lambda_l \lambda_{l'}}$ describes an intraband intralayer Coulomb interaction if $l = l'$ and an interlayer Coulomb interaction if $l \neq l'$. Furthermore, we consider the long-range part of the Coulomb interaction such that $V_{\boldsymbol{q}}^{\lambda_l \lambda'_l} \approx \frac{e_0^2}{2\epsilon_0 A|\boldsymbol{q}|\epsilon_{\text{intra},\boldsymbol{q}}}$ and $V_{\boldsymbol{q}}^{\lambda_l \lambda'_{\bar{l}}} \approx \frac{e_0^2}{2\epsilon_0 A|\boldsymbol{q}|\epsilon_{\text{inter},\boldsymbol{q}}}$ $(l \neq \bar{l})$. The intra- and interlayer dielectric functions $\epsilon_{\text{intra},\boldsymbol{q}}$ and $\epsilon_{\text{inter},\boldsymbol{q}}$ can be found in the Supplementary Material of Ref. [9]. Interband Coulomb interactions, which give rise to electron-hole exchange [10] or Auger recombination [11], are not expected to contribute significantly to experimentally accessible density-dependent energy renormalizations (Supplementary Section IV) and are therefore neglected in this work.

Given the carrier-carrier Hamiltonian, we now proceed as follows: **i)** we find the equation of motion for the intervalley polarisation $\langle P^{\dagger\xi_e l_e,\xi_h l_h}_{\boldsymbol{k}_1+\boldsymbol{Q},\boldsymbol{k}_1}\rangle \equiv \langle c^\dagger_{\xi_e,l_e,\boldsymbol{k}_1+\boldsymbol{Q}} v_{\xi_h,l_h,\boldsymbol{k}_1}\rangle$, **ii)** transform the equation of motion to the exciton basis [12], **iii)** make an ansatz for the exciton-exciton interaction Hamiltonian and compute the equation of motion for the polarisation in the exciton picture, **iv)** read off the exciton-exciton interaction matrix element such that the results from steps **ii)** and **iii)** coincide. Starting with the first step **i)**, we obtain the equation of motion for the polarisation directly from the Heisenberg equation of motion [1]. Including only the Coulomb contributions from Eq. (S7) we obtain

$$\begin{aligned} i\hbar\frac{d}{dt}\langle P^{\dagger\xi_e l_e,\xi_h l_h}_{\boldsymbol{k}_1+\boldsymbol{Q},\boldsymbol{k}_1}\rangle = \frac{1}{2}\sum_{\boldsymbol{k},\boldsymbol{q},l,\xi} \Big(& V_{\boldsymbol{q}}^{v_{l_h} v_l} (\langle c^\dagger_{\xi_e,l_e,\boldsymbol{k}_1+\boldsymbol{Q}} v_{\xi,l,\boldsymbol{k}} v_{\xi_h,l_h,\boldsymbol{k}_1-\boldsymbol{q}} v^\dagger_{\xi,l,\boldsymbol{k}-\boldsymbol{q}}\rangle - \langle c^\dagger_{\xi_e,l_e,\boldsymbol{k}_1+\boldsymbol{Q}} v_{\xi_h,l_h,\boldsymbol{k}_1-\boldsymbol{q}} v_{\xi,l,\boldsymbol{k}} v^\dagger_{\xi,l,\boldsymbol{k}-\boldsymbol{q}}\rangle) \\ & + V_{\boldsymbol{q}}^{c_l c_{l_e}} (\langle c^\dagger_{\xi,l,\boldsymbol{k}+\boldsymbol{q}} v_{\xi_h,l_h,\boldsymbol{k}_1} c^\dagger_{\xi_e,l_e,\boldsymbol{k}_1+\boldsymbol{Q}-\boldsymbol{q}} c_{\xi,l,\boldsymbol{k}}\rangle - \langle c^\dagger_{\xi_e,l_e,\boldsymbol{k}_1+\boldsymbol{Q}-\boldsymbol{q}} v_{\xi_h,l_h,\boldsymbol{k}_1} c^\dagger_{\xi,l,\boldsymbol{k}+\boldsymbol{q}} c_{\xi,l,\boldsymbol{k}}\rangle) \\ & + V_{\boldsymbol{q}}^{v_l c_{l_e}} (\langle c^\dagger_{\xi_e,l_e,\boldsymbol{k}_1+\boldsymbol{Q}-\boldsymbol{q}} v_{\xi_h,l_h,\boldsymbol{k}_1} v_{\xi,l,\boldsymbol{k}} v^\dagger_{\xi,l,\boldsymbol{k}+\boldsymbol{q}}\rangle - \langle c^\dagger_{\xi_e,l_e,\boldsymbol{k}_1+\boldsymbol{Q}-\boldsymbol{q}} v_{\xi,l,\boldsymbol{k}} v_{\xi_h,l_h,\boldsymbol{k}_1} v^\dagger_{\xi,l,\boldsymbol{k}+\boldsymbol{q}}\rangle) \\ & + V_{\boldsymbol{q}}^{v_{l_h} c_l} (\langle c^\dagger_{\xi_e,l_e,\boldsymbol{k}_1+\boldsymbol{Q}} v_{\xi_h,l_h,\boldsymbol{k}_1-\boldsymbol{q}} c^\dagger_{\xi,l,\boldsymbol{k}-\boldsymbol{q}} c_{\xi,l,\boldsymbol{k}}\rangle - \langle c^\dagger_{\xi,l,\boldsymbol{k}-\boldsymbol{q}} v_{\xi_h,l_h,\boldsymbol{k}_1-\boldsymbol{q}} c^\dagger_{\xi_e,l_e,\boldsymbol{k}_1+\boldsymbol{Q}} c_{\xi,l,\boldsymbol{k}}\rangle)\Big) . \end{aligned} \tag{S8}$$

Next, we transform the entire equation above to the excitonic basis and make use of the pair operator expansions [12]

$$c^\dagger_{\xi_e,l_e,\boldsymbol{k}} c_{\xi'_e,l'_e,\boldsymbol{k}'} \approx \sum_{\xi''_h,l''_h,\boldsymbol{k}''} P^{\dagger\xi_e l_e,\xi''_h l''_h}_{\boldsymbol{k},\boldsymbol{k}''} P^{\xi'_e l'_e,\xi''_h l''_h}_{\boldsymbol{k}',\boldsymbol{k}''} , \; v_{\xi_h,l_h,\boldsymbol{k}} v^\dagger_{\xi'_h,l'_h,\boldsymbol{k}'} \approx \sum_{\xi''_e,l''_e,\boldsymbol{k}''} P^{\dagger\xi''_e l''_e,\xi_h l_h}_{\boldsymbol{k}'',\boldsymbol{k}} P^{\xi''_e l''_e,\xi'_h l'_h}_{\boldsymbol{k}'',\boldsymbol{k}'} , \tag{S9}$$

where the pair operators can be further expressed in the exciton basis as $P^{\xi_e l_e,\xi_h l_h}_{\boldsymbol{k},\boldsymbol{k}'} = \sum_n \varphi^{\xi L}_{n,\beta^{\xi L}\boldsymbol{k}+\alpha^{\xi L}\boldsymbol{k}'} X^{\xi}_{n,L,\boldsymbol{k}-\boldsymbol{k}'}$, with $\varphi^{\xi L}_{n,\boldsymbol{k}}$ being the exciton wave function (cf. Supplementary Section I) and the compound indices $\xi = (\xi_e, \xi_h)$, $L = (l_e, l_h)$ (such that $l_e = l_h$ corresponds to the intralayer wave function and $l_e \neq l_h$ corresponds to the interlayer wave function). In the following, we will only consider the lowest-lying $n = 1s$ exciton states, so that the index n can be omitted. By doing this, the equation of motion Eq. (S8) separates into two parts, a direct part and an exchange part. The second, fourth, fifth and seventh term in Eq. (S8) gives rise to the direct terms reading

$$\begin{aligned} i\hbar\frac{d}{dt}\langle X^{\dagger\xi'}_{L',\boldsymbol{Q}}\rangle|_{\text{dir.}} = \frac{1}{2}\sum_{\boldsymbol{q},\boldsymbol{Q}_1,\xi,L} \Big(& V_{\boldsymbol{q}}^{c_{l'_e} v_{l_h}} F(\alpha^{\xi' L'}\boldsymbol{q}) F(\beta^{\xi L}\boldsymbol{q}) + V_{\boldsymbol{q}}^{c_{l_e} v_{l'_h}} F(-\alpha^{\xi' L'}\boldsymbol{q}) F(-\beta^{\xi L}\boldsymbol{q}) \\ & - V_{\boldsymbol{q}}^{c_{l_e} c_{l'_e}} F(\beta^{\xi' L'}\boldsymbol{q}) F(-\beta^{\xi L}\boldsymbol{q})) - V_{\boldsymbol{q}}^{v_{l'_h} v_{l_h}} F(-\alpha^{\xi' L'}\boldsymbol{q}) F(\alpha^{\xi L}\boldsymbol{q})\Big) \langle X^{\dagger\xi'}_{L',\boldsymbol{Q}+\boldsymbol{q}} X^{\dagger\xi}_{L,\boldsymbol{Q}_1-\boldsymbol{q}} X^{\xi}_{L,\boldsymbol{Q}_1}\rangle , \end{aligned} \tag{S10}$$

where we introduced the compound indices $\xi^{(\prime)} = (\xi^{(\prime)}_e, \xi^{(\prime)}_h)$ and $L^{(\prime)} = (l^{(\prime)}_e, l^{(\prime)}_h)$. Here, we also defined the excitonic form factors $F(x^{\xi L}\boldsymbol{q}) \equiv \sum_{\boldsymbol{k}} \varphi^{*\xi L}_{\boldsymbol{k}+x^{\xi L}\boldsymbol{q}} \varphi^{\xi L}_{\boldsymbol{k}}$. We may now construct the corresponding direct exciton-exciton interaction Hamiltonian with

$$H_{x-x}|_{\text{dir.}} = \frac{1}{2} \sum_{\substack{\boldsymbol{Q}_1,\boldsymbol{Q}_2,\boldsymbol{q} \\ \xi,\xi',L,L'}} D^{\xi\xi'}_{L,L',\boldsymbol{q}} X^{\dagger\xi'}_{L',\boldsymbol{Q}_1+\boldsymbol{q}} X^{\dagger\xi}_{L,\boldsymbol{Q}_2-\boldsymbol{q}} X^{\xi}_{L,\boldsymbol{Q}_2} X^{\xi'}_{L',\boldsymbol{Q}_1} , \tag{S11}$$

with the direct part of the exciton-exciton interaction reading

$$D^{\xi\xi'}_{L,L',\boldsymbol{q}} = \frac{1}{2}\Big(V^{c_{l_e} c_{l'_e}}_{\boldsymbol{q}} F(\beta^{\xi' L'}\boldsymbol{q}) F(-\beta^{\xi L}\boldsymbol{q})) + V^{v_{l'_h} v_{l_h}}_{\boldsymbol{q}} F(-\alpha^{\xi' L'}\boldsymbol{q}) F(\alpha^{\xi L}\boldsymbol{q}) - V^{c_{l'_e} v_{l_h}}_{\boldsymbol{q}} F(\alpha^{\xi' L'}\boldsymbol{q}) F(\beta^{\xi L}\boldsymbol{q}) - V^{c_{l_e} v_{l'_h}}_{\boldsymbol{q}} F(-\alpha^{\xi' L'}\boldsymbol{q}) F(-\beta^{\xi L}\boldsymbol{q}) \Big) , \tag{S12}$$

such that a commutation of the excitonic Hamiltonian (S11) with the polarisation gives rise to Eq. (S10). We note that, in the long wavelength limit

$$D^{\xi\xi'}_{X_i,X_i,\boldsymbol{0}} = D^{\xi\xi'}_{X_i,X_j,\boldsymbol{0}} = 0 \ , D^{\xi\xi'}_{IX_i,IX_i,\boldsymbol{0}} = -D^{\xi\xi'}_{IX_i,IX_j,\boldsymbol{0}} = \frac{e_0^2}{4A\epsilon_0}\left(\frac{d_{1,\mathrm{TMD}}}{\epsilon^{\perp}_{1,\mathrm{TMD}}} + \frac{d_{2,\mathrm{TMD}}}{\epsilon^{\perp}_{2,\mathrm{TMD}}}\right) , i \neq j \tag{S13}$$

i.e. we find a vanishing direct interaction between intralayer excitons ($L, L' = X_i$, $i = 1, 2$) and recover the widely used plate capacitor formula when considering interactions between interlayer excitons ($L, L' = IX_i$, $i = 1, 2$) as has been previously confirmed in literature [11, 13, 14]. Here, the material-specific constants $d_{i,\mathrm{TMD}}$ and $\epsilon^{\perp}_{i,\mathrm{TMD}}$ denote individual TMD monolayer thicknesses and out-of-plane components of the TMD dielectric tensors, respectively. In the main manuscript we set $d_{1,\mathrm{TMD}} = d_{2,\mathrm{TMD}} \equiv d_{\mathrm{TMD}}$ and $\epsilon^{\perp}_{1,\mathrm{TMD}} = \epsilon^{\perp}_{2,\mathrm{TMD}} \equiv \epsilon^{\perp}_{\mathrm{TMD}}$ as we are considering a homobilayer. Note that interactions between different interlayer exciton species IX_i and IX_j, $i \neq j$ are attractive due to the opposite dipole orientations of these excitons. Now, we consider the remaining terms (i.e. the first, third, fifth and eight terms) in Eq. (S8) and find that these give rise to the following exchange terms

$$\begin{aligned} i\hbar\frac{d}{dt}\langle X^{\dagger\xi'}_{L',\boldsymbol{Q}}\rangle|_{\mathrm{exch.}} = \frac{1}{2}\sum_{\substack{\boldsymbol{q},\boldsymbol{Q}_1,\boldsymbol{k},\boldsymbol{k'}\\ \xi,\tilde{\xi},\bar{\xi}\\ L,\tilde{L},\bar{L}}} \Big(&(V^{c_{l_e} c_{l'_e}}_{\boldsymbol{k}-\boldsymbol{k'}} \varphi^{\xi' L'}_{\boldsymbol{k}-\alpha^{\xi' L'}\boldsymbol{Q}-\boldsymbol{q}} - V^{c_{l_e} v_{l'_h}}_{\boldsymbol{k}-\boldsymbol{k'}} \varphi^{\xi' L'}_{\boldsymbol{k'}-\alpha^{\xi' L'}\boldsymbol{Q}-\boldsymbol{q}}) \times \\ &\delta^{\xi_h,\xi'_h}_{l_h,l'_h} \delta^{\tilde{\xi}_e,\xi'_e}_{\tilde{l}_e,l'_e} \delta^{\bar{\xi}_e,\xi_e}_{\bar{l}_e,l_e} \delta^{\bar{\xi}_h,\tilde{\xi}_h}_{\bar{l}_h,\tilde{l}_h} \varphi^{*\xi L}_{\boldsymbol{k}-\alpha^{\xi L}(\boldsymbol{Q}+\boldsymbol{q})} \varphi^{*\tilde{\xi}\tilde{L}}_{\boldsymbol{k'}-\beta^{\tilde{\xi}\tilde{L}}\boldsymbol{q}-\alpha^{\tilde{\xi}\tilde{L}}\boldsymbol{Q}_1} \varphi^{\bar{\xi}\bar{L}}_{\boldsymbol{k'}-\alpha^{\bar{\xi}\bar{L}}\boldsymbol{Q}_1} \\ &+ (V^{v_{l'_h} v_{l_h}}_{\boldsymbol{k}-\boldsymbol{k'}} \varphi^{\xi' L'}_{\boldsymbol{k}+\beta^{\xi' L'}\boldsymbol{Q}+\boldsymbol{q}} - V^{c_{l'_e} v_{l_h}}_{\boldsymbol{k}-\boldsymbol{k'}} \varphi^{\xi' L'}_{\boldsymbol{k'}+\beta^{\xi' L'}\boldsymbol{Q}+\boldsymbol{q}}) \times \\ &\delta^{\xi_e,\xi'_e}_{l_e,l'_e} \delta^{\tilde{\xi}_h,\xi'_h}_{\tilde{l}_h,l'_h} \delta^{\bar{\xi}_e,\tilde{\xi}_e}_{\bar{l}_e,\tilde{l}_e} \delta^{\bar{\xi}_h,\xi_h}_{\bar{l}_h,l_h} \varphi^{*\xi L}_{\boldsymbol{k}+\beta^{\xi L}(\boldsymbol{Q}+\boldsymbol{q})} \varphi^{*\tilde{\xi}\tilde{L}}_{\boldsymbol{k'}+\alpha^{\tilde{\xi}\tilde{L}}\boldsymbol{q}+\beta^{\tilde{\xi}\tilde{L}}\boldsymbol{Q}_1} \varphi^{\bar{\xi}\bar{L}}_{\boldsymbol{k'}+\beta^{\bar{\xi}\bar{L}}\boldsymbol{Q}_1} \Big) \times \\ &\langle X^{\dagger\xi}_{L,\boldsymbol{Q}+\boldsymbol{q}} X^{\dagger\tilde{\xi}}_{\tilde{L},\boldsymbol{Q}_1-\boldsymbol{q}} X^{\bar{\xi}}_{\bar{L},\boldsymbol{Q}_1}\rangle \end{aligned} \tag{S14}$$

from which we may construct an exchange matrix element such that

$$\frac{d}{dt}\langle X^{\dagger\xi'}_{L',\boldsymbol{Q}}\rangle|_{\mathrm{exch.}} = \frac{i}{\hbar}\sum_{\substack{\boldsymbol{q},\boldsymbol{Q}_1\\ \xi,\tilde{\xi},\bar{\xi}\\ L,\tilde{L},\bar{L}}} E^{\xi\tilde{\xi}\bar{\xi}\xi'}_{L,\tilde{L},\bar{L},L',\boldsymbol{Q},\boldsymbol{Q}_1,\boldsymbol{q}} \langle X^{\dagger\xi}_{L,\boldsymbol{Q}+\boldsymbol{q}} X^{\dagger\tilde{\xi}}_{\tilde{L},\boldsymbol{Q}_1-\boldsymbol{q}} X^{\bar{\xi}}_{\bar{L},\boldsymbol{Q}_1}\rangle , \tag{S15}$$

where the exchange part of the exciton-exciton interaction reads

$$\begin{aligned} E^{\xi_1\xi_2\xi_3\xi_4}_{L_1,L_2,L_3,L_4,\boldsymbol{Q}_1,\boldsymbol{Q}_2,\boldsymbol{q}} = \frac{1}{2}\sum_{\boldsymbol{k},\boldsymbol{k'}} \Big(&(V^{c_{l_{1,e}} v_{l_{4,h}}}_{\boldsymbol{k}-\boldsymbol{k'}} \varphi^{\xi_4 L_4}_{\boldsymbol{k'}-\alpha^{\xi_4 L_4}\boldsymbol{Q}_1-\boldsymbol{q}} - V^{c_{l_{1,e}} c_{l_{4,e}}}_{\boldsymbol{k}-\boldsymbol{k'}} \varphi^{\xi_4 L_4}_{\boldsymbol{k}-\alpha^{\xi_4 L_4}\boldsymbol{Q}_1-\boldsymbol{q}}) \times \\ &\delta^{\xi_{1,h},\xi_{4,h}}_{l_{1,h},l_{4,h}} \delta^{\xi_{2,e},\xi_{4,e}}_{l_{2,e},l_{4,e}} \delta^{\xi_{3,e},\xi_{1,e}}_{l_{3,e},l_{1,e}} \delta^{\xi_{3,h},\xi_{2,h}}_{l_{3,h},l_{2,h}} \varphi^{*\xi_1 L_1}_{\boldsymbol{k}-\alpha^{\xi_1 L_1}(\boldsymbol{Q}_1+\boldsymbol{q})} \varphi^{*\xi_2 L_2}_{\boldsymbol{k'}-\beta^{\xi_2 L_2}\boldsymbol{q}-\alpha^{\xi_2 L_2}\boldsymbol{Q}_2} \varphi^{\xi_3 L_3}_{\boldsymbol{k'}-\alpha^{\xi_3 L_3}\boldsymbol{Q}_2} \\ &+ (V^{c_{l_{4,e}} v_{l_{1,h}}}_{\boldsymbol{k}-\boldsymbol{k'}} \varphi^{\xi_4 L_4}_{\boldsymbol{k'}+\beta^{\xi_4 L_4}\boldsymbol{Q}_1+\boldsymbol{q}} - V^{v_{l_{4,h}} v_{l_{1,h}}}_{\boldsymbol{k}-\boldsymbol{k'}} \varphi^{\xi_4 L_4}_{\boldsymbol{k}+\beta^{\xi_4 L_4}\boldsymbol{Q}_1+\boldsymbol{q}}) \times \\ &\delta^{\xi_{1,e},\xi_{4,e}}_{l_{1,e},l_{4,e}} \delta^{\xi_{2,h},\xi_{4,h}}_{l_{2,h},l_{4,h}} \delta^{\xi_{3,e},\xi_{2,e}}_{l_{3,e},l_{2,e}} \delta^{\xi_{3,h},\xi_{1,h}}_{l_{3,h},l_{1,h}} \varphi^{*\xi_1 L_1}_{\boldsymbol{k}+\beta^{\xi_1 L_1}(\boldsymbol{Q}_1+\boldsymbol{q})} \varphi^{*\xi_2 L_2}_{\boldsymbol{k'}+\alpha^{\xi_2 L_2}\boldsymbol{q}+\beta^{\xi_2 L_2}\boldsymbol{Q}_2} \varphi^{\xi_3 L_3}_{\boldsymbol{k'}+\beta^{\xi_3 L_3}\boldsymbol{Q}_2} \Big) . \end{aligned} \tag{S16}$$

Here, we note that the first term corresponds to hole-hole exchange within the excitons and the second term corresponds to electron-electron exchange. In particular, the Kronecker deltas imply that fermionic exchange of individual charge constituents is only allowed if charges of the same species reside in the same layer and valley. Moreover, the exchange interaction is generally dependent on both centre-of-mass momenta $\boldsymbol{Q}_1, \boldsymbol{Q}_2$ as well as the relative momentum $\boldsymbol{q}$. In the long wavelength limit ($q, Q_1, Q_2 \ll a_B^{-1}$, a_B being the exciton Bohr radius) the exchange interaction is non-vanishing for both intra- and interlayer exciton species, and it is the dominating contribution to the exciton-exciton

interaction for intralayer excitons [13, 15, 16]. However, we remark that the resulting density-dependent energy renormalizations due to intralayer exchange interactions are negligible (see Supplementary Section IV). Exchange interactions are therefore not considered in the main manuscript, but included here only for the sake of completeness. Hence, we obtain the exchange part of the exciton-exciton Hamiltonian

$$H_{x-x}|_{\text{exch.}} = \frac{1}{2} \sum_{\substack{\boldsymbol{Q}_1,\boldsymbol{Q}_2,\boldsymbol{q} \\ \xi_1...\xi_4 \\ L_1...L_4}} E^{\xi_1\xi_2\xi_3\xi_4}_{L_1,L_2,L_3,L_4,\boldsymbol{Q}_1,\boldsymbol{Q}_2,\boldsymbol{q}} X^{\dagger\xi_1}_{L_1,\boldsymbol{Q}_1+\boldsymbol{q}} X^{\dagger\xi_2}_{L_2,\boldsymbol{Q}_2-\boldsymbol{q}} X^{\xi_3}_{L_3,\boldsymbol{Q}_2} X^{\xi_4}_{L_4,\boldsymbol{Q}_1} \ . \tag{S17}$$

We now have access to the multilayer exciton-exciton interaction involving both intra- and interlayer excitons. However, generally, excitons are hybridized between the layers due to electron/hole tunneling. To include the effect of hybridization, we transform the excitonic operators to the hybrid basis, cf. Eq. (S4). The exciton-exciton Hamiltonian then transforms into a hybrid Hamiltonian

$$\tilde{H}_{x-x} = \frac{1}{2} \sum_{\substack{\eta_1...\eta_4 \\ \xi_1...\xi_4 \\ \boldsymbol{Q}_1,\boldsymbol{Q}_2,\boldsymbol{q}}} \tilde{W}^{\xi_1\xi_2\xi_3\xi_4}_{\eta_1,\eta_2,\eta_3,\eta_4,\boldsymbol{Q}_1,\boldsymbol{Q}_2,\boldsymbol{q}} Y^{\dagger\xi_1}_{\eta_1,\boldsymbol{Q}_1+\boldsymbol{q}} Y^{\dagger\xi_2}_{\eta_2,\boldsymbol{Q}_2-\boldsymbol{q}} Y^{\xi_3}_{\eta_3,\boldsymbol{Q}_2} Y^{\xi_4}_{\eta_4,\boldsymbol{Q}_1} \ , \tag{S18}$$

where the hybrid exciton-exciton interaction contains of a direct part and an exchange part according to

$$\tilde{D}^{\xi_1\xi_2}_{\eta_1,\eta_2,\eta_3,\eta_4,\boldsymbol{Q}_1,\boldsymbol{Q}_2,\boldsymbol{q}} = \sum_{L_1,L_2} D^{\xi_1\xi_2}_{L_1,L_2,\boldsymbol{q}} C^{*\xi_1\eta_1}_{L_1,\boldsymbol{Q}_1+\boldsymbol{q}} C^{\xi_1\eta_4}_{L_1,\boldsymbol{Q}_1} C^{*\xi_2\eta_2}_{L_2,\boldsymbol{Q}_2-\boldsymbol{q}} C^{\xi_2\eta_3}_{L_2,\boldsymbol{Q}_2} \ , \tag{S19}$$

and

$$\tilde{E}^{\xi_1\xi_2\xi_3\xi_4}_{\eta_1,\eta_2,\eta_3,\eta_4,\boldsymbol{Q}_1,\boldsymbol{Q}_2,\boldsymbol{q}} = \sum_{L_1,L_2,L_3,L_4} E^{\xi_1\xi_2\xi_3\xi_4}_{L_1,L_2,L_3,L_4,\boldsymbol{Q}_1,\boldsymbol{Q}_2,\boldsymbol{q}} C^{*\xi_1\eta_1}_{L_1,\boldsymbol{Q}_1+\boldsymbol{q}} C^{\xi_4\eta_4}_{L_4,\boldsymbol{Q}_1} C^{*\xi_2\eta_2}_{L_2,\boldsymbol{Q}_2-\boldsymbol{q}} C^{\xi_3\eta_3}_{L_3,\boldsymbol{Q}_2} \ , \tag{S20}$$

with the unhybrised direct (D) and exchange (E) matrix elements defined in Eq. (S12) and (S16), respectively. Having derived the most general form of the hybrid exciton-exciton interaction, we now remark on the considered case of untwisted homobilayers. In this case, it holds that the mixing coefficients are approximately constant in momentum, such that $C^{\xi\eta}_{L,\boldsymbol{Q}} \approx C^{\xi\eta}_{L}$ [4]. Consequently, the direct hybrid exciton-exciton interaction depends only on the relative momentum $\boldsymbol{q}$. In the main manuscript, we only consider the lowest-lying hybrid exciton states for each valley configuration and therefore the indices η_i, $i = 1...4$ are omitted therein. Moreover, note that the intra- and interlayer mixing coefficients enter the hybrid exciton-exciton interaction strengths. This provides an intriguing way of tuning the interaction strength with externally applied electric fields.

III. DIPOLE-DIPOLE INTERACTION

In here, we show that the real-space representation of the direct interlayer exciton-exciton interaction (Eq. (S12), with $L = L' = IX$ and $\xi = \xi'$) can be interpreted as a classical dipole-dipole interaction at large distances. By considering the two TMD layers forming the homobilayer as two infinitely thin slabs separated by the distance d and approximating the dielectric environment as homogenous, with a single effective dielectric constant ϵ_{BL}, we find an analytic expression for the direct exciton-exciton interaction. Within these approximations, the intra (X)- and interlayer (IX) Coulomb interactions read [3]

$$V^X_{\boldsymbol{q}} = \frac{e_0^2}{2\epsilon_0 A|\boldsymbol{q}|\epsilon_{\text{BL}}} \ , V^{IX}_{\boldsymbol{q}} = \frac{e_0^2}{2\epsilon_0 A|\boldsymbol{q}|\epsilon_{\text{BL}}(1+\tanh(|\boldsymbol{q}|d))} \ . \tag{S21}$$

Now, substituting these simplified expressions into the direct exciton-exciton interaction and setting the excitonic form factors $F \approx 1$, we find that the direct interlayer exciton-exciton interaction reduces to

$$D_{IX,IX,\boldsymbol{q}} \approx \frac{e_0^2}{2\epsilon_0 A|\boldsymbol{q}|\epsilon_{\text{BL}}}(1 - \frac{1}{1+\tanh(|\boldsymbol{q}|d)}) \ . \tag{S22}$$

The real-space representation of the interaction is obtained by taking the Fourier-transform:

$$D_{IX,IX}(\boldsymbol{r}) = \frac{e_0^2}{4\pi\epsilon_0\epsilon_{\text{BL}}}(\frac{1}{|\boldsymbol{r}|} - \frac{1}{d(\sqrt{4+|\boldsymbol{r}|^2/d^2})}) \ . \tag{S23}$$

Finally, we are interested in the asymptotic behavior of the interaction and therefore let $r \gg d$. In this limit, we find

$$D_{IX,IX}(\boldsymbol{r}) = \frac{d^2 e_0^2}{2\pi\epsilon_0\epsilon_{\rm BL}} \frac{1}{|\boldsymbol{r}|^3} + \mathcal{O}(|\boldsymbol{r}|^{-5}) \ , \tag{S24}$$

which is precisely a classical dipole-dipole interaction. Now, as the interlayer components of the mixing coefficients are approximately constant in momentum (cf. Supplementary Section I) for untwisted TMD homobilayers, we note that also the corresponding real-space hybrid exciton-exciton interaction obeys the asymptotic $1/r^3$ behavior for large r. In Fig. S1, we show the direct hybrid exciton-exciton interaction strength between KΛ excitons in hBN-encapsulated WSe_2 homobilayers as a function of distance, r, including the dominant interlayer contributions to the interaction (solid yellow curve). Unlike in the main manuscript, we here plot the logarithm of the interaction strength over large distances. Importantly, we find that the interaction scales as $1/r^3$ (dashed black curve) at distances $r \gtrsim 15$ nm. This confirms the dipole-dipole-like character of the hybrid exciton-exciton interaction at large distances.

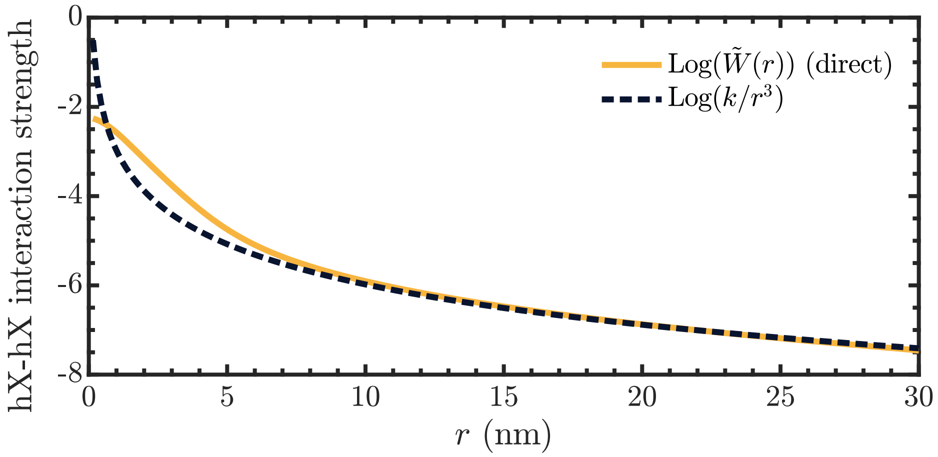

FIG. S1. Real-space representation of the hybrid exciton-exciton interaction for KΛ excitons. Note that we here have taken the logarithm of the interaction strength.

IV. DENSITY-DEPENDENT ENERGY RENORMALIZATIONS FOR HYBRID EXCITONS

Having access to the microscopic hybrid exciton-exciton Hamiltonian (Eq. (S18) and Supplementary Section II) implies that we are now also able to investigate density-dependent energy renormalizations of hybrid excitons. These energy renormalizations can be derived from the corresponding Heisenberg equation of motion for the (hybrid) polarisation $\langle Y^\dagger_{\zeta,\boldsymbol{Q}}\rangle$ reading

$$\frac{d}{dt}\langle Y^\dagger_{\zeta,\boldsymbol{Q}}\rangle = \frac{i}{\hbar}\sum_{\boldsymbol{Q}_1,\boldsymbol{q},\zeta_1,\zeta_2,\zeta_3} \tilde{W}^{\zeta_1\zeta_2\zeta_3\zeta}_{\boldsymbol{Q},\boldsymbol{Q}_1,\boldsymbol{q}} \langle Y^\dagger_{\zeta_1,\boldsymbol{Q}+\boldsymbol{q}} Y^\dagger_{\zeta_2,\boldsymbol{Q}_1-\boldsymbol{q}} Y_{\zeta_3,\boldsymbol{Q}_1}\rangle \ , \tag{S25}$$

where we introduced the compound index $\zeta = (\xi, \eta)$ including the valley index ξ and the hybrid exciton index η. Now, we consider the equation above on a Hartree-Fock level, i.e. we expand the appearing bosonic three-operator expectation value into single-particle expectation values and neglect two-particle correlations. We also make use of the random phase approximation (RPA) [1] such that the equation above becomes

$$\frac{d}{dt}\langle Y^\dagger_{\zeta,\boldsymbol{Q}}\rangle \approx \frac{i}{\hbar}\sum_{\boldsymbol{q},\zeta_1}(\tilde{W}^{\zeta\zeta_1\zeta_1\zeta}_{\boldsymbol{Q},\boldsymbol{q},0} + \tilde{W}^{\zeta_1\zeta\zeta_1\zeta}_{\boldsymbol{Q},\boldsymbol{q},\boldsymbol{q}-\boldsymbol{Q}}) N^{\zeta_1}_{\boldsymbol{q}} \langle Y^\dagger_{\zeta,\boldsymbol{Q}}\rangle \ , \tag{S26}$$

where we defined the hybrid exciton occupation $N^\zeta_{\boldsymbol{Q}} \equiv \langle Y^\dagger_{\zeta,\boldsymbol{Q}} Y_{\zeta,\boldsymbol{Q}}\rangle$. Here we also approximated $\langle Y^\dagger_{\zeta,\boldsymbol{Q}} Y_{\zeta',\boldsymbol{Q}'}\rangle \approx \delta^{\zeta,\zeta'}_{\boldsymbol{Q},\boldsymbol{Q}'} N^\zeta_{\boldsymbol{Q}}$ making use of the RPA. Note that the energy renormalization consists of two terms, the first term being a direct term and the second being an exchange term, with the latter reflecting exciton exchange [13]. In contrast to the exchange interaction $\tilde{E}$ which includes exchange of individual carriers, the exciton exchange takes into account the exchange of

individual excitons. Furthermore, we consider low temperatures in this work such that the exciton distribution $N_{\boldsymbol{q}}$ is strongly peaked around $\boldsymbol{q} = 0$ and assume the centre-of-mass momentum $|\boldsymbol{Q}| \ll a_B^{-1}$, where a_B is the exciton Bohr radius. This reduces the equation above to $\frac{d}{dt}\langle Y^\dagger_{\zeta,\boldsymbol{Q}}\rangle \approx \frac{i}{\hbar}\delta E^\zeta \langle Y^\dagger_{\zeta,\boldsymbol{Q}}\rangle$ introducing the energy renormalization

$$\delta E^\zeta = A \sum_{\zeta_1} (\tilde{W}^{\zeta_1\zeta\zeta_1\zeta}_{0,0,0} + \tilde{W}^{\zeta\zeta_1\zeta_1\zeta}_{0,0,0}) n_x^{\zeta_1} \ , \tag{S27}$$

where $n_x^{\zeta_1} \equiv \frac{1}{A}\sum_{\boldsymbol{Q}} N^{\zeta_1}_{\boldsymbol{Q}}$ with A being the crystal area (cancelling out with the area A in the electronic Coulomb matrix elements). Finally, we restrict ourselves to the energetically lowest hybrid exciton states in this work such that the compound index ζ reduces to the valley index ξ. Generally, the energy renormalization of a hybrid exciton ξ is obtained by taking into account the interactions between all the different exciton species. By assuming that $n_x^\xi \propto n_x$, i.e. assuming a thermalized Boltzmann distribution for the hybrid excitons, where $n_x = \sum_\xi n_x^\xi$ is the total exciton density, the energy renormalization of a single exciton species can be quantified by a valley-specific effective dipole length d^ξ obtained from

$$\delta E^\xi = A \sum_{\xi_1} (\tilde{W}^{\xi_1\xi\xi_1\xi}_{0,0,0} + \tilde{W}^{\xi\xi_1\xi_1\xi}_{0,0,0}) n_x^{\xi_1} \equiv \frac{d^\xi e_0^2}{\epsilon_0 \epsilon^\perp_{\text{TMD}}} n_x \ . \tag{S28}$$

In the evaluation of valley-specific dipole lengths we here only include the direct (dipole-dipole) contributions to the hybrid exciton-exciton interaction, cf. Eq. (S18). This is done as interlayer exchange interactions are seen to provide a small quantitative correction to the dipole-dipole interaction [9]. Moreover, although intralayer exchange interactions taking into account individual exchange of carriers are dominant in TMD monolayers [11, 14], they have negligible impact on the energy renormalizations, as their contributions are largely cancelled out against contributions due to higher-order correlations such as biexcitons [10]. This goes beyond the scope of the Hartree-Fock theory presented in this work.

In Fig. S2 we illustrate the valley-specific effective dipole length as obtained from Eq. (S28) for the case of naturally stacked hBN-encapsulated WSe_2 homobilayers as a function of electric field, E_z at low temperatures, $T = 10$ K. Intriguingly, we find a drastic increase in the dipole length at around $E_z \pm 0.15$ V/nm. This reflects the transition from a mostly intralayer KΛ (K'Λ') state to a mostly interlayer KΛ' (K'Λ) state under the application of a positive (negative) electric field. At vanishing electric fields, KΛ and K'Λ' excitons coexist. These excitons independently interact via weak repulsive dipole-dipole interactions and mutually interact with each other via attractive dipolar interactions as they exhibit opposite dipole orientations, giving rise to negligible effective dipole lengths. At the largest considered electric fields, $E_z = 0.3$ V/nm, only KΛ' excitons are relevant and the impact of other excitons is negligible due to the large energy separations between exciton states (cf. Table S1). These excitons are mostly interlayer-like in nature ($|C_{IX}|^2 = 0.8$, cf. Table S1) and interact strongly via dipole-dipole repulsion. Note that the large effective dipole moment of KΛ' excitons directly translates into large energy renormalizations (Eq. (S28)) and give rise to sizable blue-shifts of phonon sidebands with exciton density, as schematically illustrated by the inset in Fig. S2.

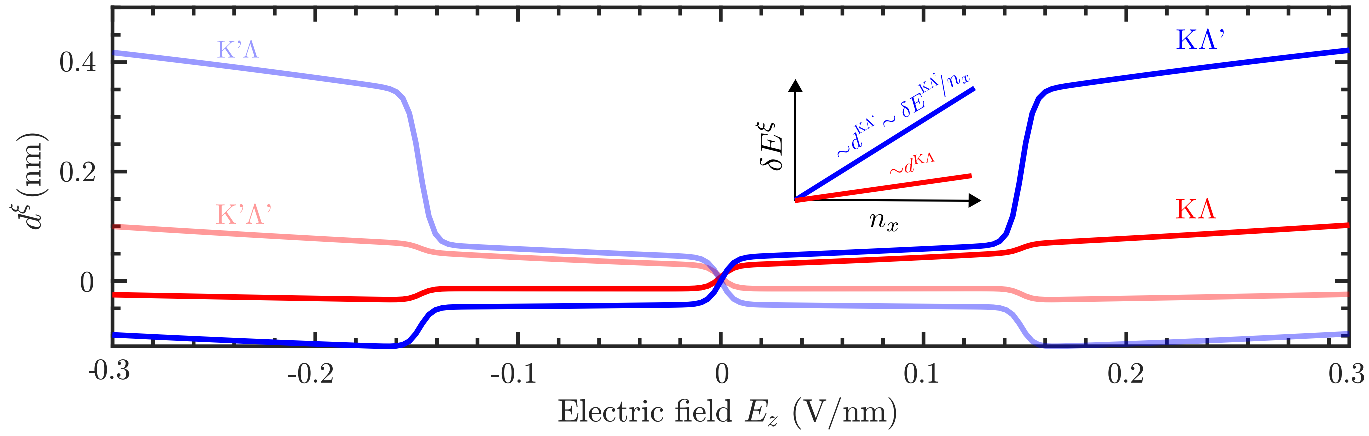

FIG. S2. Valley-specific effective dipole length in naturally stacked hBN-encapsulated WSe_2 homobilayers. The valley-specific dipole length d^ξ determines the corresponding energy renormalization $\delta E^\xi \sim d^\xi n_x$ for an individual exciton species ξ (cf. inset).

[1] M. Kira and S. W. Koch, Many-body correlations and excitonic effects in semiconductor spectroscopy, Progress in quantum electronics **30**, 155 (2006).

[2] A. Kormányos, G. Burkard, M. Gmitra, J. Fabian, V. Zólyomi, N. D. Drummond, and V. Fal'ko, k· p theory for two-dimensional transition metal dichalcogenide semiconductors, 2D Materials **2**, 022001 (2015).

[3] S. Ovesen, S. Brem, C. Linderälv, M. Kuisma, T. Korn, P. Erhart, M. Selig, and E. Malic, Interlayer exciton dynamics in van der Waals heterostructures, Communications Physics **2**, 1 (2019).

[4] J. Hagel, S. Brem, C. Linderälv, P. Erhart, and E. Malic, Exciton landscape in van der Waals heterostructures, Physical Review Research **3**, 043217 (2021).

[5] S. Brem, K.-Q. Lin, R. Gillen, J. M. Bauer, J. Maultzsch, J. M. Lupton, and E. Malic, Hybridized intervalley moiré excitons and flat bands in twisted WSe_2 bilayers, Nanoscale **12**, 11088 (2020).

[6] S. Brem, C. Linderälv, P. Erhart, and E. Malic, Tunable phases of moiré excitons in van der Waals heterostructures, Nano Letters **20**, 8534 (2020).

[7] A. Laturia, M. L. Van de Put, and W. G. Vandenberghe, Dielectric properties of hexagonal boron nitride and transition metal dichalcogenides: from monolayer to bulk, npj 2D Materials and Applications **2**, 1 (2018).

[8] J. Hagel, S. Brem, and E. Malic, Electrical tuning of moiré excitons in mose2 bilayers, 2D Materials **10**, 014013 (2022).

[9] D. Erkensten, S. Brem, R. Perea-Causín, and E. Malic, Microscopic origin of anomalous interlayer exciton transport in van der Waals heterostructures, Physical Review Materials **6**, 094006 (2022).

[10] F. Katsch, M. Selig, and A. Knorr, Theory of coherent pump–probe spectroscopy in monolayer transition metal dichalcogenides, 2D Materials **7**, 015021 (2019).

[11] D. Erkensten, S. Brem, and E. Malic, Exciton-exciton interaction in transition metal dichalcogenide monolayers and van der Waals heterostructures, Physical Review B **103**, 045426 (2021).

[12] F. Katsch, M. Selig, A. Carmele, and A. Knorr, Theory of exciton–exciton interactions in monolayer transition metal dichalcogenides, physica status solidi (b) **255**, 1800185 (2018).

[13] C. Ciuti, V. Savona, C. Piermarocchi, A. Quattropani, and P. Schwendimann, Role of the exchange of carriers in elastic exciton-exciton scattering in quantum wells, Physical Review B **58**, 7926 (1998).

[14] V. Shahnazaryan, I. Iorsh, I. A. Shelykh, and O. Kyriienko, Exciton-exciton interaction in transition-metal dichalcogenide monolayers, Physical Review B **96**, 115409 (2017).

[15] C. Schindler and R. Zimmermann, Analysis of the exciton-exciton interaction in semiconductor quantum wells, Physical Review B **78**, 045313 (2008).

[16] F. Tassone and Y. Yamamoto, Exciton-exciton scattering dynamics in a semiconductor microcavity and stimulated scattering into polaritons, Physical Review B **59**, 10830 (1999).
